# Human Echolocators Have Better Localization Off Axis

**DOI:** 10.1177/09567976211068070

**Published:** 2022-06-14

**Authors:** Lore Thaler, L. J. Norman, H. P. J. C. De Vos, D. Kish, M. Antoniou, C. J. Baker, M. C. J. Hornikx

**Affiliations:** 1 Department of Psychology, Durham University; 2 Department of the Built Environment, Eindhoven University of Technology; 3 World Access for the Blind, Placentia, California; 4 Department of Electronic Electrical and Systems Engineering, University of Birmingham

**Keywords:** blindness, hearing, psychophysics, behavior, bats

## Abstract

Here, we report novel empirical results from a psychophysical experiment in which we tested the echolocation abilities of nine blind adult human experts in click-based echolocation. We found that they had better acuity in localizing a target and used lower intensity emissions (i.e., mouth clicks) when a target was placed 45° off to the side compared with when it was placed at 0° (straight ahead). We provide a possible explanation of the behavioral result in terms of binaural-intensity signals, which appear to change more rapidly around 45°. The finding that echolocators have better echo-localization off axis is surprising, because for human source localization (i.e., regular spatial hearing), it is well known that performance is best when targets are straight ahead (0°) and decreases as targets move farther to the side. This may suggest that human echolocation and source hearing rely on different acoustic cues and that human spatial hearing has more facets than previously thought.

Echolocation is the ability to use reflected sound to obtain information about the spatial environment. It is probably best known from bats and marine mammals, but people can employ echolocation as well, for example, by using mouth clicks (Kolarik et al., 2014; Thaler & Goodale, 2016). Previous studies have measured the acuity of human echolocation using mouth clicks for localization of objects positioned straight ahead in front of the echolocator (Teng et al., 2012; Thaler et al., 2011); however, there has been no attempt to measure acuity at other angles. Rowan et al. (2015) have suggested that echolocation is based on binaural-intensity cues, yet their important work was based on localization straight ahead, using white-noise emissions and a passive-listening paradigm. Interestingly, optimum received intensity for the human ear and head is at around 45° azimuth (Fortune, 1997; Shaw, 1974; Shaw & Vaillancourt, 1985). Therefore, if the *intensity hypothesis* (Rowan et al., 2015) applies to active echolocation using mouth clicks, human echolocation should be better for localizing objects placed 45° off to the side, compared with straight ahead (0°) and with farther angles (e.g., 90°).

We here tested this novel prediction in a psychophysical experiment with a group of nine expert echolocators. Participants’ task was to first echolocate the target at a reference position, which could be 0° (straight ahead), 45°, or 90° azimuth. The different reference positions were tested in separate blocks. Second, participants echolocated the target at a comparison position, which could be either clockwise or counterclockwise with respect to the reference; the exact position on every trial was determined by an adaptive staircase procedure. Participants then had to judge whether the target was located clockwise or counterclockwise with respect to the reference position.

It has been shown previously that human echolocators may dynamically adjust the intensity and number of mouth clicks to compensate for weaker echoes (Thaler et al., 2018, 2019). Thus, we might also expect a change in the clicks that people make for targets placed off to the side, and to that end, we also measured people’s mouth clicks while they performed the task.

## Method

### Ethics statement

The experiment was conducted following the British Psychological Society code of practice and according to the World Medical Association Declaration of Helsinki. All procedures were approved by the Durham University Department of Psychology ethics committee (Ref. No. 14/13). Participants volunteered to take part in the study. Information and consent forms were provided in an accessible format, and we obtained informed consent from all participants. No observations were excluded from data analysis.

### Participants

Nine participants who were blind and used echolocation on a daily basis took part in the experiment. We used convenience sampling, and sample size was determined by the availability of participants. At this point, there are only a few people who use click-based echolocation regularly, so we had some practical limitations. Our sample size (*N* = 9) was similar to or exceeded sample sizes in other reports about click-based echolocation.

Any participants who had eyes were blindfolded during testing. Participant 1 (male, 32 years old) has had the ability to detect only bright light since age 8 as a result of optic-nerve atrophy. He has been using click-based echolocation since age 29. Participant 2 (male, 53 years old) has total blindness in the right eye and the ability to detect only bright light in the left eye since age 5 as the result of optic-nerve compression. He has been using click-based echolocation since age 43. Participant 3 (female, 41 years old) has been totally blind since birth as the result of Leber’s congenital amaurosis. She has been using click-based echolocation since age 31. Participant 4 (male, 49 years old) has been totally blind since age 1 as the result of retinoblastoma and subsequent enucleation. He has been using click-based echolocation for as long as he can remember. Participant 5 (male, 33 years old) has been totally blind since age 14 as the result of optic-nerve atrophy. He has been using click-based echolocation since age 15. Participant 6 (male, 56 years old) has had the ability to detect only bright light since birth as the result of retinal detachment. He has been using click-based echolocation since age 6. Participant 7 (male, 43 years old) has had the ability to detect only bright light in the right eye and total blindness in the left eye from birth as a result of Leber’s congenital amaurosis. He has been using click-based echolocation since age 33. Participant 8 (male, 34 years old) is totally blind and experienced gradual vision loss since birth as the result of glaucoma. He has been using click-based echolocation since age 12. Participant 9 (female, 40 years old) has been totally blind since age 22 months as the result of retinoblastoma and subsequent enucleation. She has been using click-based echolocation since age 31.

**Statement of Relevance**

Bats and dolphins are well known for their ability to use echolocation. They make bursts of sounds and listen to the echoes that bounce back. People can employ echolocation as well, for example, by using mouth clicks. In our study, we tested the echolocation abilities of blind adults who had experience in click-based echolocation. We found that they were better at localizing targets placed 45° off to the side, compared with targets placed 0° straight ahead. We provide a possible explanation based on the intensity of echoes and how this differs between the ears. The finding that echolocators have better echo-localization off axis is surprising, because for regular spatial hearing, localization is best when targets are straight ahead. Our results suggest that human spatial hearing has more facets than previously thought. Also, echolocation provides real-life advantages for people with vision impairments. Thus, this work is of interest to users of echolocation and to people providing instruction.

All except Participant 2 had normal hearing, as assessed with pure-tone audiometry (500–8000 Hz). Participant 2 had hearing loss (~15 dB) from 500 to 4000 Hz.

### Setup and apparatus

All testing was conducted in a 2.9 m × 4.2 m × 4.9 m noise-insulated and echo-dampened room (walls were lined with foam wedges with a cutoff frequency of 315 Hz, and the floor was covered with foam baffles; the room had a noise floor of 24 dBA). Participants stood in the center of the room. Tactile markers were used to allow participants to reliably place their heads without impeding movements of the mouth for clicking. The target was a wooden disk (17.5 cm in diameter and 5 mm thick) presented at a distance of 100 cm from the participant on top of a steel pole (0.5 cm in diameter). The center of the target was at mouth level, and the target always faced the participant. The floor had marks for positioning of the target with 0.1° precision. We made recordings of testing sessions with a digital recorder (TASCAM DR100-MKII recorder; TEAC, Japan; 24 bit and 96 kHz) and microphones (DPA SMK-SC4060 miniature microphones, 4 mm in diameter; DPA Microphones, Allerød, Denmark). Microphones were placed on either side of the participant’s head, slightly in front and on top of the tragus. Stimulus presentation and behavioral and acoustic analyses were done using MATLAB (The MathWorks, Natick, MA) and custom-written routines.

### Procedure

On any given trial, the target was presented twice: first at the reference position, which could be 0° (straight ahead), 45°, or 90° azimuth (always on the left side; this was done for practical reasons, so that testing did not take too long). Second, the target was presented at the comparison position, which could be either clockwise or counterclockwise with respect to the reference. The comparison position on every trial was determined using an adaptive staircase procedure (see below). Participants’ task was to first echolocate the target at the reference position. Subsequently, they blocked their ears, and the experimenter repositioned the target to the comparison position (this took around 30 s). Once this had been done, the experimenter tapped the participants, who then unblocked their ears and echolocated the target. They then had to judge whether the target had shifted clockwise or counterclockwise in relation to the reference position. To make sure that participants understood the task, we provided alternative descriptions and response options; for example, right versus left for the central testing location, toward the periphery (e.g., on an arc from the participants’ straight ahead toward a more eccentric location), or toward the center (e.g., on an arc from the participants’ sides toward their straight ahead). Participants were free to perform as many practice trials as they wanted. Testing commenced only when it was clear that the participants understood what was asked of them and that they were confident with the response options. While participants made clicks, the experimenter sat on the floor behind them.

To minimize the possibility of procedural bias, we used two intertwined staircases that approached the reference position clockwise or counterclockwise, each starting from a 36° angular difference from the reference position. Presentation order of staircases was pseudorandomized such that one staircase would not run for more than four consecutive trials. The angular difference between test and reference on each trial was determined adaptively. In the first two trials, we used the stochastic approximation by Robbins and Monro (1951):

$$x_{n+1}=x_{n}-\frac{c}{n(z_{n}-\varphi )},$$

where *n* is the number of the current trial, *x* the value of the stimulus, *c* the initial step size (set at 36°), and ϕ the probability of responding in a correct or an incorrect way with respect to the corresponding staircase (0.5 in our paradigm); *z* defines whether the response was correct (1) or incorrect (0), referring to the corresponding staircase (e.g., “clockwise” was correct for the clockwise-starting staircase and incorrect for the counterclockwise-starting staircase). For subsequent trials, we used the accelerated stochastic approximation by Kesten (1958), which can be used as an extension of the original stochastic approximation provided by Robbins and Monro (1951):

$$x_{n+1}=x_{n}–\frac{c}{\left(2+m)(z_{n}-\varphi \right)}.$$

This equation additionally includes *m* for the number of changes in the response category—that is, *m* increased by 1 when the response switched from clockwise to counterclockwise, or vice versa, in one staircase.

Each test took at most 45 min to complete. Participants took breaks between tests. The order in which locations were tested changed across participants.

### Psychophysical data analysis

Psychophysical performance was measured by fitting two-parameter sigmoid curves of the form

$$F=\frac{1}{1+\exp\left(-\frac{x-a}{b}\right)}$$

to data for each reference position separately. This was done using the lsqcurvefit.m function implemented in MATLAB performing a nonlinear least-squares fit with a trust-region algorithm. To compute thresholds, we first determined those points on the curve where the probability of judging a stimulus as clockwise was either .25 or .75. We then computed the average of the absolute threshold values. To compute bias, we determined the point on the curve where the probability of judging a reflector as clockwise was .5.

### Acoustical analyses of mouth clicks

To characterize each participant’s clicking behavior, we analyzed recorded sound files. We analyzed the number of clicks made for each trial, click duration, click intensity, interclick intervals, and click power spectra, as well as power-spectral centroid and bandwidth based on power spectra. The number of clicks for each trial was determined visually and aurally by analyzing the sound files. During this process, clicks were also isolated from intermittent speech and other background noise (e.g., coughing, swallowing) for further analysis. Click duration was computed as the time from click onset to offset. To obtain onset and offset, we first computed the click envelope as the absolute value of the signal and smoothed it with a moving average using a 0.42-ms-duration window. Click onset was determined as the first point where envelope value exceeded 5% (−26 dB) of the maximum. Click offset was determined by fitting a decaying exponential to the envelope (starting from envelope maximum; using the lsqcurvefit.m function implemented in MATLAB performing a nonlinear least-squares fit with a trust-region algorithm). The offset was then defined as the point where the fitted curve dropped to 5% of the maximum. Click intensity was computed as root-mean-square intensity of clicks. To characterize the spectral content of clicks, we computed each click’s power spectrum and then determined the power-spectral centroid and the bandwidth for each trial using a 25-dB drop relative to peak (Arditi et al., 2015) and using the powerbw.m function implemented in the MATLAB signal-processing toolbox. We then averaged across trials for each location.

### Acoustical analyses of HRTF data

To investigate binaural-intensity changes as a function of azimuth angle, we analyzed previously published head-related transfer function (HRTF) measurements (Austrian Academy of Sciences Institute for Sound Research, 2020). Although this database contains data from 220 individual HRTF measurements, we analyzed only 97 HRTFs that had been obtained with microphones inside the ear canal. HRTFs had been measured with a frequency sweep from 200 to 16000 Hz (for more details, see Austrian Academy of Sciences Institute for Sound Research, n.d.). We analyzed only HRTFs at 0° elevation and in two 5° steps around each testing location (i.e., for 0° straight ahead: −10°, −5°, 0°, 5°, 10°; for 45° left: 35°, 40°, 45°, 50°, 55°; for 90° left: 80°, 85°, 90°, 95°, 100°). For each angle and participant, we calculated the average absolute binaural-level difference across 2 to 10 kHz between the left and right channel. This value was then averaged across participants. A parallel analysis was performed for spectral bands 1 to 10 kHz and 4 to 10 kHz. Another parallel analysis was performed for timing cues (i.e., binaural-timing differences), in which we calculated phase differences between right and left channels. Calculations were performed using MATLAB.

### Statistical data analyses

To investigate effects of the different conditions on thresholds, bias, and clicking behavior, we subjected data to a repeated measures analysis of variance (ANOVA). Post hoc pairwise comparisons were done using two-tailed, paired-samples *t* tests. For all analyses, statistical significance was determined using a threshold (α) of .05, Bonferroni-corrected for multiple comparisons where appropriate (Bonferroni-corrected α = .0167). Greenhouse-Geisser (GG) correction was applied if the sphericity assumption could not be upheld. Statistical analyses were carried out in SPSS (Version 26).

## Results

**Fig. 1.**
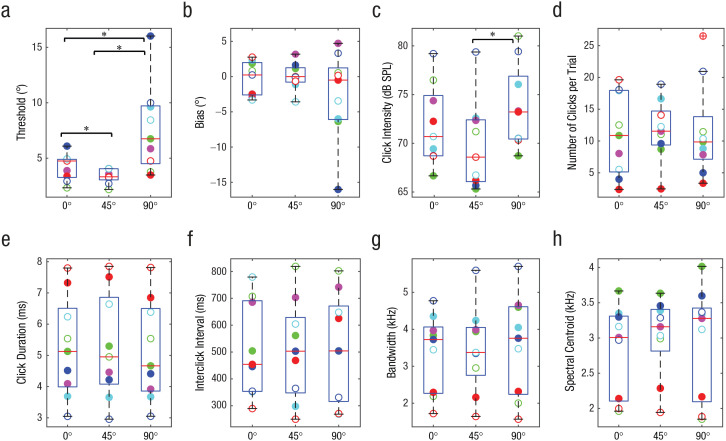
Measures of echolocation behavior. The graphs show (a) localization threshold, (b) bias, (c) click intensity, (d) number of clicks, (e) click duration, (f) interclick interval, (g) bandwidth (25-dB drop relative to peak), and (h) power spectral centroid, separately for targets placed at 0°, 45°, and 90° relative to the position of participants. In each box-and-whisker plot, the red horizontal bar indicates the median, and the lower and upper boundaries of the box mark the 25th and 75th percentiles, respectively; whiskers extend to 1.5 times the interquartile range, drawn back to the closest data point. Circles denote data from individual participants (a separate color is used consistently for each participant across graphs, and solid and empty circles correspond to the solid and dashed lines in Fig. 2). Asterisks indicate significant differences between target locations (*p < .05, Bonferroni corrected), as determined by paired-samples *t* tests. SPL = sound-pressure level.

Individually fitted psychometric functions are provided in Figure S1 in the Supplemental Material available online. Figure 1a shows participants’ localization thresholds separately for the 0°, 45°, and 90° reference positions. Lower thresholds indicate that people can discriminate smaller changes in azimuth (i.e., better performance). Overall, people performed remarkably well: Average thresholds for targets placed at 0°, 45°, and 90° were 4.2°, 3.4°, and 7.6°, respectively, and individual thresholds were as low as 2.2°. Importantly, participants did better (i.e., had lower thresholds) at 45° compared with 0°, and performance was worst at 90°. Consistent with these observations, a repeated measures ANOVA with location (0°, 45°, and 90°) as a repeated variable showed a significant effect of location on thresholds, *F*_GG_(2, 8.36) = 10.73, *p* = .010, η_*p*_^2^ = .573, power = .831. Pairwise comparisons were significant for 0° versus 45°, *t*(8) = 3.45, *p* = .009, mean difference = 0.850°, 95% confidence interval (CI) = [0.281, 1.419], *r* = .830; for 45° versus 90°, *t*(8) = 3.42, *p* = .009, mean difference = −4.255°, 95% CI = [−7.123, −1.387], *r* = .412; and for 0° versus 90°, *t*(8) = 3.07, *p* = .015, mean difference = −3.405°, 95% CI = [−5.961, −0.849], *r* = .637.

Figure 1b shows participants’ bias separately for the 0°, 45°, and 90° reference positions. It is evident that bias was close to zero at all reference positions. Consistent with these observations, a repeated measures ANOVA with location as a repeated variable showed no effect of location on bias, *F*(2, 16) = 1.142, *p* = .344, η_*p*_^2^ = .125, power = .216, and one-sample *t* tests comparing bias with zero were not significant for any reference position—0°: *t*(8) = 0.43, *p* = .677; 45°: *t*(8) = 0.055, *p* = .958; 90°: *t*(8) = 1.25, *p* = .247.

Figure 1c shows the intensity of people’s clicks, and it is evident that it changed across testing locations and that click intensity was lowest at 45° and highest at 90°. Consistent with these observations, a repeated measures ANOVA with location as a repeated variable showed a significant effect of location on click intensity (in decibels of sound-pressure level [db SPL]), *F*(2, 16) = 8.73, *p* = .003, η_*p*_^2^ = .522, power = .934. Pairwise comparisons were significant for 45° versus 90°, *t*(8) = 3.83, *p* = .005, mean difference = 3.891 db SPL, 95% CI = [1.554, 6.230], *r* = .768, but not for 0° versus 90°, *t*(8) = 2.29, *p* = .051, mean difference = 1.835 db SPL, 95% CI = [0.010, 3.681], *r* = .839, or for 0° versus 45°, *t*(8) = 2.12, *p* = .066, mean difference = 2.057 db SPL, 95% CI = [0.176, 4.289], *r* = .783.

**Fig. 2.**
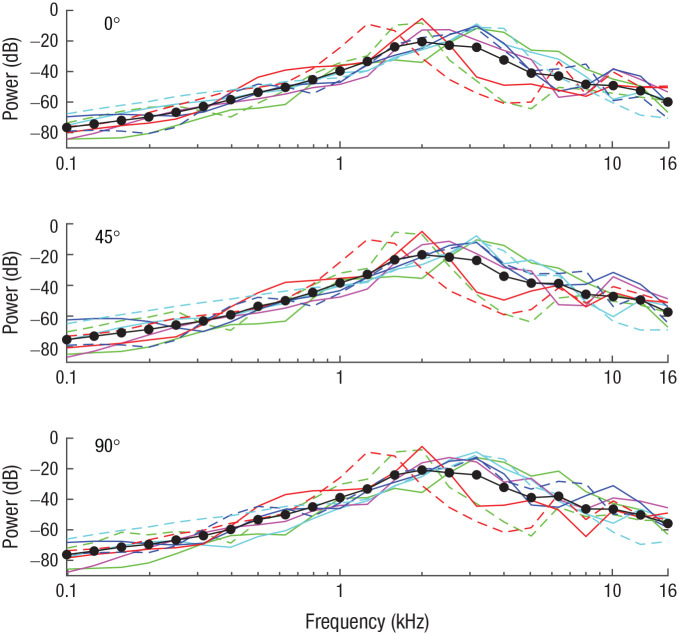
Click power spectra (1/3 octave bands with respect to total power) for the 0°, 45°, and 90° testing locations (top, middle, and bottom graphs, respectively). A separate line color and type is used across all three graphs for each of the nine participants, and the solid and dashed lines correspond to the solid and empty circles, respectively, in Figure 1. Black lines and symbols denote averages across participants.

None of the other aspects of mouth clicks (i.e., number of clicks, click duration, interclick interval, bandwidth, and spectral centroid) changed across testing locations (Figs. 1d–1h). Click power spectra also remained unchanged (Fig. 2). This finding is in agreement with those of previous studies showing no change in click power spectra (or spectral centroid or bandwidth) as a function of task demands (e.g., Thaler et al., 2018, 2019). Yet the current study is the first to provide such data in the context of a localization task.

**Fig. 3.**
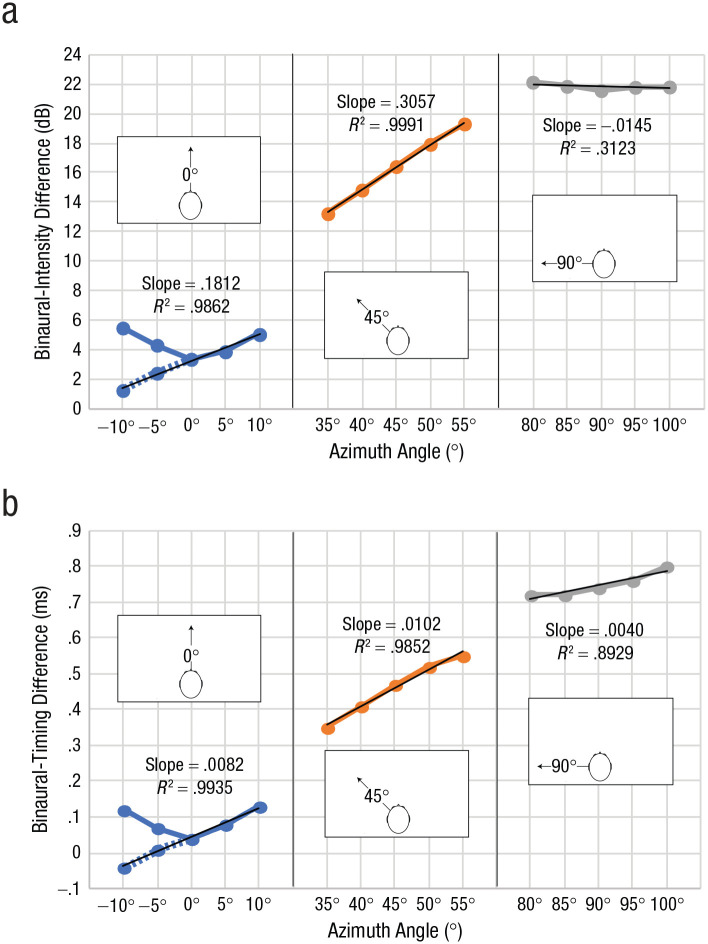
Characterization of level and timing cues for the 2- to 10-kHz frequency range. Binaural-intensity changes (a) and binaural-timing changes (b) are shown as a function of azimuth angle, separately for the 0°, 45°, and 90° positions of the target, respectively. U-shaped curves result from intensity being calculated as an absolute (i.e., unsigned) measure. For each reference location, we used the difference between each spatial location (azimuth angle) and the next to calculate linear regression slopes. Values for –10° and –5° azimuth angle have been flipped (shown as dashed lines) to facilitate this analysis at the 0° reference position.

It has been suggested that people use binaural-intensity signals above 2 kHz to echolocate target azimuths (Rowan et al., 2015). Although it is well known that the human ear and head have an optimum in terms of received intensity around 45° azimuth (Fortune, 1997; Shaw, 1974; Shaw & Vaillancourt, 1985), there has been no specific analysis to determine whether this also translates into binaural cues. Hence, we calculated binaural cues on the basis of previously published HRTF measurements (http://sofacoustics.org/data/database/ari/). HRTFs provide individualized measurements of sound intensity, timing, and spectrum for any ear and location measured. Thus, they can be used to investigate how various aspects of sound change across space. Figure 3a shows binaural-intensity changes (for the 2- to 10-kHz frequency range) as a function of angle in 5° steps separately for 0°, 45°, and 90° azimuth positions, respectively. U-shaped curves at azimuth 0° result from intensity being calculated as an absolute (i.e., unsigned) measure. This does not necessarily imply that they are less informative.

To investigate how binaural-intensity differences change as a function of spatial position for each reference location, we used the difference between each spatial location and the next to calculate linear regression slopes. To facilitate this analysis at the 0° reference position, we flipped values for −10° and −5° (shown as dashed lines in Fig. 3a). To investigate slope changes statistically, we conducted a regression analysis in which binaural differences in intensity were treated as the dependent variable and angular differences (−10°, −5°, 0°, 5°, 10°), testing location (0°, 45°, 90°, dummy-coded with 0° as the reference category), and the interaction between these two variables were treated as predictors. A significant interaction term between reference location and angular differences would indicate that slopes differed across testing locations, and the beta weight tells us whether the slope is higher (i.e., positive β weight) or lower (i.e., negative β weight) than the slope at 0°. The regression model overall fitted the data well, *R* = .999, *F*(5, 9) = 6,822.450, *p* < .001. The constant was significantly higher than zero, β = 3.183, *t*(9) = 42.575, *p* < .001. Yet, as is also evident in Figure 3a, the mean difference was higher at 45° compared with 0°, β = 13.180, *t*(9) = 124.663, *p* < .001, and also higher at 90° than at 0°, β = 18.681, *t*(9) = 176.697, *p* < .001. Overall, there was a positive slope (i.e., a positive relationship between angular difference and binaural-intensity changes), β = 0.181, *t*(9) = 17.143, *p* < .001. But, most important, the slope at 45° was significantly higher than slope at 0°, β = 0.125, *t*(9) = 8.327, *p* < .001, and at 90° it was significantly lower, β = −0.196, *t*(9) = −13.09, *p* < .001. The results illustrate that binaural-intensity differences as a function of angle change most rapidly around the 45° reference position and more slowly around the 0° and 90° reference positions. Thus, smaller differences in azimuth position around 45° led to larger differences in binaural-intensity differences compared with other positions. This might be a possible explanation for our behavioral results. Specifically, if people rely on binaural-intensity signals to determine target azimuth, then they may have an easier time detecting a change in azimuth when there is a larger corresponding change in binaural intensity.

We also performed a parallel analysis for binaural-timing differences, and the results are shown in Figure 3b. The regression model overall fitted the data well, *R* = .999, *F*(5, 9) = 2386.861, *p* < .001. The constant was significantly higher than zero, β = 0.044, *t*(9) = 9.429, *p* < .001. Yet, as is also evident in Figure 3b, the mean difference was higher at 45° compared with 0°, β = 0.416, *t*(9) = 63.034, *p* < .001, and also higher at 90° than at 0°, β = 0.704, *t*(9) = 106.672, *p* < .001. Overall there was a positive slope (i.e., a positive relationship between angular difference and binaural-timing changes), β = 0.008, *t*(9) = 12.425, *p* < .001. But, most important, the slope at 45° was not significantly higher than the slope at 0°, β = 0.002, *t*(9) = 2.143, *p* = .061, and at 90° it was significantly lower, β = −0.004, *t*(9) = −4.5, *p* = .001. The results suggest that binaural-timing differences also change as a function of angle but that, in contrast to intensity differences, the rate of change is similar around 0° and 45° but lower at 90°. Thus, timing cues are present alongside intensity cues, but binaural-intensity differences show a more distinct advantage in terms of rate of change around 45°.

**Fig. 4.**
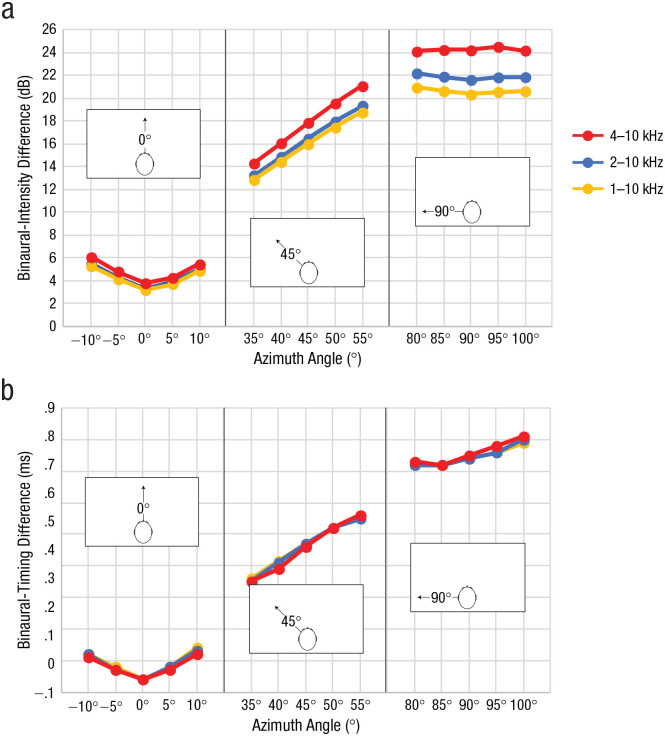
Characterization of level and timing cues for different spectral frequencies. Binaural-intensity changes (a) and binaural-timing changes (b) are shown as a function of azimuth angle and spectral-frequency cutoff, separately for the 0°, 45°, and 90° positions of the target, respectively.

HRTF measurements were done with frequency sweeps (200–16000 Hz), and our results are therefore not limited to any specific sound. But the following is relevant to demonstrate that our acoustic analysis applies to sounds with the unique profile of echoes. Echoes will contain spectral frequencies contained in the clicks (shown in Fig. 2), but they will also show an attenuation of lower spectral frequencies because of the size of the reflector (in our study, a 17.5-cm diameter). The reason is that the size of the reflector strongly attenuates reflections for sound below a certain limit (i.e., for frequencies for which the product of the wave number and the reflector radius is smaller than 1; in our case, 1200 Hz), because these essentially wash over or bend around the object (e.g., Kuttruff, 2007). Thus, we performed our acoustic analyses for different cutoffs for the lower frequency range. The results are shown in Figure 4.

With respect to binaural intensity (Fig. 4a), the results show that the advantage around 45° was always there but that it became more pronounced for sound with higher frequency content. This suggests not only that the pattern of results we observed is generally valid but also that it is particularly pronounced for sounds with the unique profile of echoes. This is unlike binaural changes in timing (Fig. 4b), which are largely independent from frequency.

## Discussion

Our novel empirical findings show that echolocation performance improves when targets are placed at 45° relative to the listener, compared with 0° and 90°. At 45°, people also exhibit the lowest click intensity, and this finding is consistent with previous observations that people may decrease click intensity when received echo signals are stronger (Thaler et al., 2018, 2019). Although both timing and intensity cues are available to participants to perform the task, our acoustic analyses and previous literature (Rowan et al., 2015) suggest that changes in binaural-intensity signals might be the most likely explanation for our behavioral results. In this way, our novel empirical result is consistent with a novel prediction based on the intensity hypothesis by Rowan et al. (2015), suggesting that this hypothesis also holds when people with long-term experience in echolocation via mouth clicks actively use this skill.

The behavioral and acoustic results presented here were obtained from two separate data sets—that is, from our participant sample and a separate HRTF database. It would be interesting to see whether future work could provide more direct evidence (e.g., if differences between participants’ HRTFs could explain differences in their spatial sensitivity).

The task we used is the echoic equivalent of minimum-audible-angle tasks, which have a long tradition in auditory research measuring people’s ability to localize sound sources (e.g., see Blauert, 1997, for a textbook, or Battal et al., 2020, and the literature cited therein, for recent research reports involving people with blindness). These sorts of tasks require participants to judge the location of a comparison sound with respect to a reference sound. Importantly, because both sounds are presented in isolation and with a temporal gap in between (in our paradigm, the time gap between presentations was around 30 s because the stimulus had to be physically placed), one must perceptually resolve the location of each individual sound being presented in order to perform the task. Therefore, perceptual thresholds in minimum-audible-angle tasks are taken as measurements of people’s ability to localize sound. Importantly, even though the reference remains constant throughout a test, the comparison stimulus changes from trial to trial, and in our paradigm, it changed even more so because we used an adaptive method. Thus, we cannot think of a specific stimulus-response mapping or strategy that participants could apply across trials.

The data were obtained in a group of blind expert echolocators who performed the experiment under acoustically controlled conditions and while keeping their heads stable. This was required for us to understand which acoustic cues are relevant. In daily life, echolocators are faced with a great array of external sounds, and they move their heads when performing echolocation. Although people can also perform echolocation in the presence of background noise, future research is needed to address how the results we found here may generalize under such conditions. For example, a prediction following from our findings is that echolocators using clicks in a scenario in which they can move their heads may orient their heads slightly away from the target when their goal is to localize the target’s position.

Also, in bats, it has been proposed that localization via echolocation is better off axis (i.e., away from straight ahead). In bats, however, this effect was explained by angular changes in intensity in the emission, that is, on the basis of characteristics of the transmitter (Yovel et al., 2010). In contrast, here we explain the results on the basis of the human ear (i.e., characteristics of the receiver). Importantly, the underlying principle is the same: Localization performance is based on changes in signal intensity (i.e., echo). This suggests that there are common aspects of sensory processing for echolocation in bats and people, which is remarkable considering the vast differences in signal design and auditory systems.

Importantly, for source localization (i.e., regular spatial hearing), performance decreases from 0° to 45° to 90°, and it is well known that it relies on both binaural-intensity and binaural-timing differences (Blauert, 1997; King et al., 2001). Consequently, the effect we found here may demonstrate that localization via echolocation and localization through source hearing may place different emphases on different acoustic cues and could therefore be thought of as following different principles, suggesting that human spatial hearing has more facets than previously thought.

Using biological systems such as bats and people will be helpful for developing low-cost (i.e., not array-based) artificial radar and sonar systems, because understanding their sensing principles can serve as an inspiration for these systems that are just emerging in research (Smith et al., 2016). Working with humans in this context can facilitate instructions and measurements.

Furthermore, echolocation is a skill that provides real-life advantages for people who are blind (Norman et al., 2021; Thaler, 2013). Learning about echolocation and the characteristics of human performance will be useful for new users and for people providing instruction and information.

## Supplemental Material

sj-pdf-1-pss-10.1177_09567976211068070 – Supplemental material for Human Echolocators Have Better Localization Off Axis Supplemental material, sj-pdf-1-pss-10.1177_09567976211068070 for Human Echolocators Have Better Localization Off Axis by Lore Thaler, L. J. Norman, H. P. J. C. De Vos, D. Kish, M. Antoniou, C. J. Baker and M. C. J. Hornikx in Psychological Science

## Transparency

*Action Editor:* Krishnankutty Sathian

*Editor:* Patricia J. Bauer

*Author Contributions*

L. Thaler developed the study concept and designed the experiment. D. Kish contributed to the study design. Testing and data collection were performed by L. Thaler, H. P. J. C. De Vos, and L. J. Norman. L. Thaler analyzed the behavioral and acoustic click data. M. C. J. Hornikx analyzed the head-related transfer function (HRTF) data. L. Thaler drafted the manuscript, and L. J. Norman, M. C. J. Hornikx, M. Antoniou, C. J. Baker, and D. Kish provided critical revisions. All the authors approved the final manuscript for submission.

**Open Practices:** Data and materials for this study have not been made publicly available, and the design and analysis plans were not preregistered.
