# Supplementary material for: Human Echolocators Have Better Localization Off Axis
Source: Psychol Sci. 2022 Jun 14;33(7):1143–53. doi: 10.1177/09567976211068070 (PMC13020946; doi:10.1177/09567976211068070)
Supplement: sj-pdf-1-pss-10.1177_09567976211068070 – Supplemental material for Human Echolocators Have Better Localization Off Axis [file sj-pdf-1-pss-10.1177_09567976211068070.pdf]

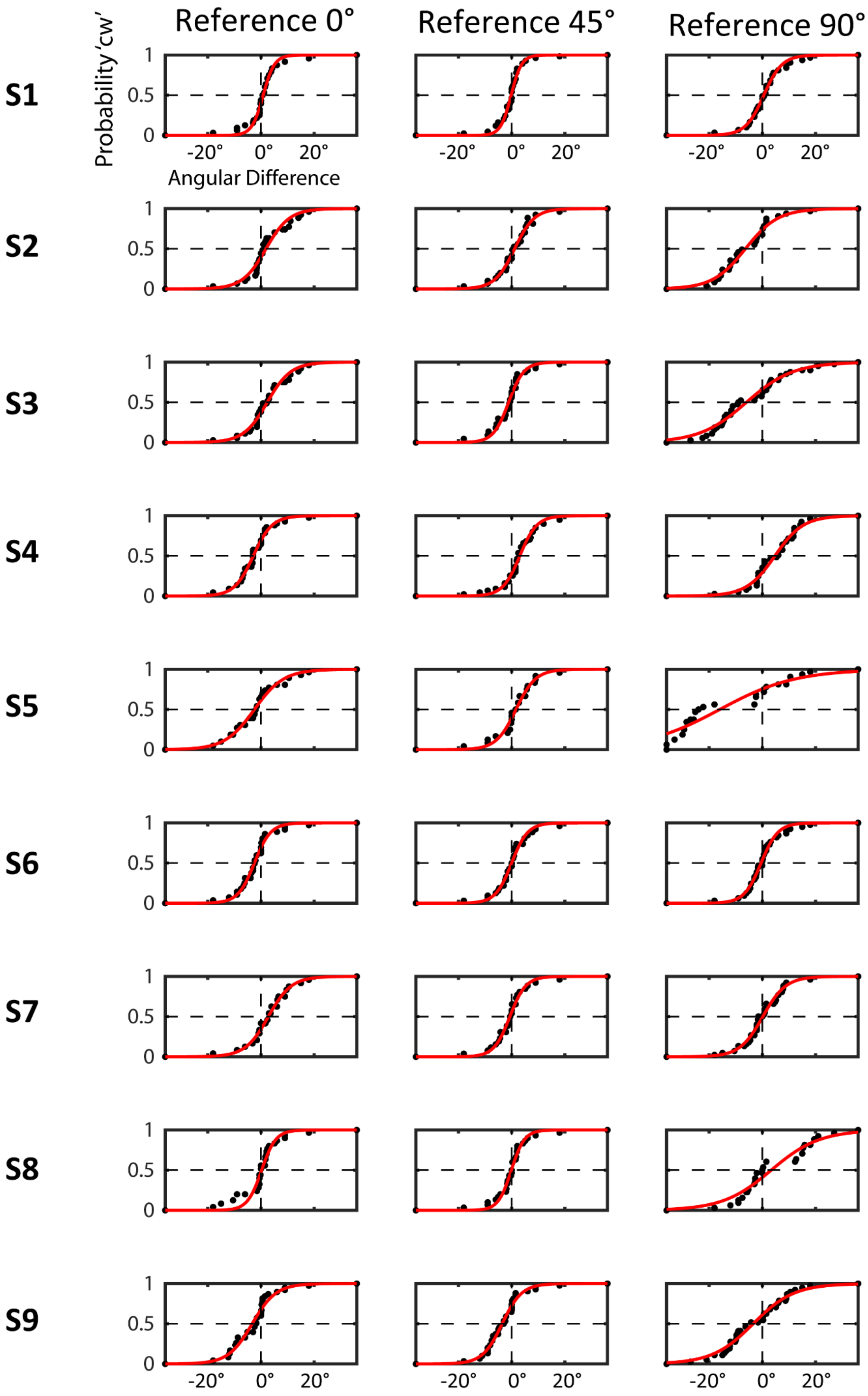

Supplementary Figure S1 – Individual psychometric data. Black dots are data points and red curves are fitted psychometric functions. Dashed horizontal and vertical lines are drawn for reference. Different rows show data for different participants (S1-S9, corresponding to labels in the main text). Different columns show data for different testing location (0˚, 45˚, 90˚). In each individual plot, the x-axis shows the angular difference between the reference and the comparison stimulus in degrees. The y-axis shows the probability for the location of the comparison to be judged clockwise compared to the location of the reference.
